# COVID-19 Vaccine Booster Hesitancy among the Elderly in Malaysian Residential Care Homes: A Cross-Sectional Study in Klang Valley

**DOI:** 10.3390/vaccines12030268

**Published:** 2024-03-04

**Authors:** Kai Wei Lee, Sook Fan Yap, Hooi Tin Ong, Sien Leong Liew, Myo Oo, Kye Mon Min Swe

**Affiliations:** 1 Department of Medical Microbiology, Faculty of Medicine and Health Sciences, Universiti Putra Malaysia, Serdang 43400, Malaysia; lee_kai_wei@yahoo.com; 2 Department of Pre-Clinical Sciences, M. Kandiah Faculty of Medicine and Health Sciences, Universiti Tunku Abdul Rahman, Kajang 43000, Malaysia; onght@utar.edu.my; 3 School of Business and Economics, Universiti Putra Malaysia, Serdang 43400, Malaysia; liewsienleong@gmail.com; 4 Department of Population Medicine, M. Kandiah Faculty of Medicine and Health Sciences, Universiti Tunku Abdul Rahman, Kajang 43000, Malaysia; myo@utar.edu.my; 5 School of Medicine, Newcastle University Medicine Malaysia, Iskandar Puteri 79200, Malaysia; kye-mon.min-swe@newcastle.edu.my

**Keywords:** elderly, COVID-19 vaccine, booster hesitancy, residential care home

## Abstract

The elderly are considered a high-risk group for severe outcomes and death from COVID-19 infection. Given the emergence of new COVID variants and the immunity provided by vaccines waning over time, booster doses of the vaccine have been advocated for those at risk to stay protected. This study aimed to determine the factors associated with hesitancy toward the second booster of the COVID-19 vaccine among the elderly residing in residential care homes. A cross-sectional study was conducted in 24 residential care homes in the Klang Valley using a face-to-face interview questionnaire. The study population included individuals aged 60 and above who had been fully vaccinated against COVID-19 up to the first booster dose. Second-booster hesitancy was assessed using the Oxford Vaccine Hesitancy Scale with seven items, the aggregate score of which ranges from seven to thirty-five; the higher the score, the greater the level of hesitancy. Multivariate linear regression was employed to determine factors associated with second-booster hesitancy, and a *p*-value < 0.05 was considered statistically significant. Data from 401 elderly individuals were included for analysis. The mean score of the Oxford Vaccine Hesitancy Scale was 21.6 ± 7.2. Predictors of second booster hesitancy were identified. Age, Indian ethnicity, being a recipient of the Sinovac vaccine as the first COVID-19 booster, experiencing the death of close friends or immediate family members following COVID-19 vaccination, and negative messages (indicating that taking a booster dose is harmful) from caregivers, friends, or family members were found to be associated with an increased second-booster-hesitancy score. Conversely, positive messages (indicating that taking a booster is helpful) from the government and caregivers, friends, or family members were identified as predictors associated with a reduction in the second-booster-hesitancy score. While vaccines effectively combat severe COVID-19, the majority of the elderly hesitate before taking the second booster. Their hesitancy, rooted in the perception of a low self risk and reliance on protection from the initial doses, emphasizes the need for intervention by relevant bodies. Taking into consideration the risk, albeit relatively low, of potentially serious side effects following COVID-19 vaccinations, it is imperative that transparent, appropriate, and positive messaging regarding booster vaccines, particularly in the context of the elderly from residential care homes, be available. Encouraging this high-risk group to embrace the second booster aligns with the goal of maximizing protection within the vulnerable elderly population.

## 1. Introduction

The COVID-19 pandemic has presented unprecedented challenges globally [1], especially for the elderly population, who are particularly vulnerable due to their heightened risk of adverse outcomes including severe or critical complications requiring intensive care and assisted ventilation, complicated disease course, single or multiple organ failure, and systemic inflammatory response [2,3]. The elderly comprise a high-risk group compared to the young and middle-aged, given their higher rates of comorbidities and decreased immune function [4]. The mortality rate follows a concerning trajectory, escalating from 35 deaths per 100,000 COVID-19 cases at 60 years of age through 70 deaths per 100,000 cases among those aged 70, to 110 deaths per 100,000 cases among those aged 80 [5]. This underscores the critical importance of addressing the need to protect the elderly population in the context of COVID-19 infection.

Arising from these concerns, global initiatives have been implemented to safeguard the vulnerable, including elderly people, from COVID-19. This includes prioritizing them to receive the initial vaccine dose and subsequent booster shots [6,7]. As of October 2022, the vaccination progress portal of the Ministry of Health Malaysia indicated that approximately 69% of the Malaysian adult population had received at least one dose of a booster [8]. However, overall, the booster coverage for the whole population stood at 50.6% which is much lower compared to other countries such as Italy with 80.9, and Peru with 93.3 [9]. Other efforts included a national surveillance system that informs individuals and their dependents about vaccination appointment scheduling and permits active reporting of adverse events associated with the COVID-19 vaccine [10,11]. 

Numerous studies that focus on the elderly have identified several factors associated with hesitancy toward receiving the booster dose of the COVID-19 vaccine, such as a self-perceived low susceptibility to COVID-19 [12], perceived barriers to obtaining the booster [12,13], a perceived lack of benefits from receiving the booster dose [12], and having a negative attitude toward vaccines [14]. Other factors reported include the belief that the first or second dose of COVID-19 vaccine is sufficient [15], prior receipt of the Sinovac COVID-19 vaccine [15], experiencing side effects [15], the perception that the vaccine is ineffective in preventing COVID-19 [15], and facing “vaccine fatigue” due to receiving repeated COVID-19 vaccination [13,15]. In addition, demographic background, such as being female [13] and a younger age [16,17] appear to play a role in shaping attitudes towards COVID-19 booster vaccination. It has also been noted that personal issues such as forgetfulness and lack of time contribute to compliance failure [15].

A number of investigations have explored the perception of the COVID-19 vaccine boosters among the elderly [12,14,15,16,17,18,19,20]. However, research specifically addressing hesitancy toward a second booster among the elderly is limited [13]. Moreover, there is a notable scarcity of literature that explicitly delves into the elderly in residential care homes. This group of elderly people may receive comparatively less attention from their family members and encounter reduced interaction with the general public in contrast to those living with family or independently. Being isolated in care homes for extended periods significantly restricts their access to up-to-date information about the emergence of new COVID variants, government directives for follow-up second booster doses, and the overall status of COVID-19. Their primary sources of information include television, radio, smartphones, social groups within the care home, and interaction with caregivers. 

Although their exposure to the general public is limited, thus the risk of contracting COVID-19 is presumably lower, the focus on the elderly in residential care homes lies in their increased susceptibility to severe infections and outcomes. Therefore, understanding their hesitancy towards receiving a second booster is pivotal for shaping effective public health interventions, even though they may not be the primary focus in the broader community. In this study, we aim to determine factors associated with hesitancy towards the second COVID-19 vaccine booster among the elderly in Malaysian residential care homes in the Klang Valley region where the infection rate has been among the highest. The anticipated outcome is the formulation of strategies that promote informed decisions about vaccine uptake in this target population.

## 2. Materials and Methods

### 2.1. Study Design, Sampling Method and Inclusion Criteria

This was a cross-sectional study using a face-to-face interview questionnaire involving elderly people (≥aged 60 years) from Malaysian residential care homes in the Klang valley region. In Malaysia, the definition of “elderly” is a person aged 60 years or over [21,22]. Prior to the start of data collection, ethical clearance (U/SERC/119/2022) was obtained from the UTAR Scientific and Ethical Review Committee through the institutional review board. 

Random sampling using an online randomizer was employed to select residential care homes (RCHs) from a list of 158 facilities, of which 75 were selected. The inclusion of these care homes was determined by chance, ensuring each care home had an equal opportunity for selection. Consent for the survey was successfully obtained from 24 RCHs. All elderly individuals within these homes were enrolled in the study using universal sampling, contingent upon their voluntary agreement and satisfaction of specific inclusion criteria. Data were collected from April 2023 to September 2023.

To be eligible, individuals had to meet the following inclusion criteria: (i) Malaysian nationality, (ii) 60 years of age or older, (iii) informed consent given, and (iv) completed the first and second dose and first booster dose of the COVID-19 vaccine. Individuals were excluded if they were bedridden or had cognitive challenges that hindered their ability to make informed decisions.

### 2.2. Instruments and Variables

The Oxford COVID-19 Vaccine Hesitancy Scale, consisting of 7 items, was adapted from Freeman et al. [23], with a minor modification involving the addition of “second booster.” This adjustment ensured the continued relevance of the questions in assessing hesitancy towards the COVID-19 vaccine second booster. The scoring method remained the same, ranging from 1 (indicating acceptance of the second booster) to 5 (indicating rejection of the second booster), and the “I don’t know” option was been removed from the scoring. The scores of the 7 items were summed to derive an aggregate score, where a higher score on the Oxford COVID-19 Vaccine Hesitancy Scale reflects greater hesitancy towards the second booster.

For the independent variables, the study encompasses various factors including age, gender, ethnicity, education level, the presence of chronic illnesses, being on medication for chronic conditions, a history of COVID-19 infection, and prior hospitalization due to COVID-19. Additionally, the research involved inquiries into participants’ self-reported information and that of their immediate family or close friends regarding mild or severe/critical reactions following doses 1 and 2, as well as the first booster. Concerns after receiving the first booster dose were also explored. The investigation further delved into the types of information individuals have received from government sources, social media, telecommunication apps, caregivers, close friends, and immediate family members.

### 2.3. Statistical Analysis

The IBM SPSS Statistics software package for Windows (Version 21) was used for data analysis. Descriptive analyses were presented as n values (%) for categorical independent variables and as mean ± standard deviation for continuous data. The independent samples *t*-test was employed to compare the mean scores of Oxford COVID-19 Vaccine Hesitancy between two groups of independent variables, Pearson correlation coefficient was used to assess the linear relationship between two continuous variables, and a one-way ANOVA was applied if there were more than two groups of independent variables. Variables with a *p*-value less than 0.05 were deemed statistically significant in both the Student’s *t*-test and ANOVA tests. Variables with a *p*-value < 0.25 in the bivariate analyses were selectively included in the multivariate linear regression analysis. This decision was made because opting solely for variables with *p*-values < 0.05 in the bivariate analysis could potentially overlook variables that are recognized as important but may not meet the stringent significance threshold [24]. The enter model was used in multivariate linear regressions to identify predictors of hesitancy toward the second booster of the COVID-19 vaccine. In the final regression model, variables with a *p*-value < 0.05 were considered statistically significant. This approach ensures a comprehensive exploration of potential predictors while still adhering to the standard significance threshold of <0.05 in the multivariate analysis.

## 3. Results

### 3.1. Characteristics of Participants and Factors Associated with Vaccine Hesitancy on Bivariate Analysis

This study included 401 participants in the analysis (Table 1). The mean age was 70.5 years; an association between age and hesitancy score using the Pearson test demonstrated a significant correlation between these two parameters (r = 0.191, *p* < 0.001). The participants were also divided into four age groups as shown in Table 1 and statistical analysis likewise demonstrated a significant association between age and hesitancy score (*p* = 0.007). In addition, the Tukey HSD post hoc test was applied, which showed that the differences in the mean hesitancy scores across the various age groups were statistically significant. More than half were males (59.6%) and about two-thirds were Chinese (66.6%), with the majority having a chronic illness (80.3%). 

With respect to COVID-19 infection, 79.3% had never been infected; of the remaining people who had been infected (n = 83, 20.7%), only 19 (4.7%) were hospitalized. Notably, hospitalization due to COVID-19 infection was significantly associated with a higher hesitancy score (*p* = 0.037). Post vaccination, 30.2% and 27.4% experienced mild reactions following the first/second or first booster doses of the COVID-19 vaccine, respectively, while 1.7% and 2% had severe/critical reactions following the first/second dose or the first booster, respectively. There was a statistically significant association between serious side effects post-vaccination after the first/second dose and hesitancy (*p* = 0.004). However, there was no such association with the first booster; instead, mild reactions after the first booster appeared to have a significant, albeit weaker, association (*p* = 0.039). 

Additionally, 15.2% were concerned that the first booster might cause serious long-term side effects, while 2.7% were concerned that the protective effects of the first booster might not last that long. The reported rate of hospitalization was 8.2% and the rate of death was 8.5% among their close friends/family members following COVID-19 vaccination. It is noted that none of these factors had an association with hesitancy for the second booster. Instead, negative messages about the booster dose, most commonly heard from caregivers/friends/family members (11.2%), followed by social media/telecommunication apps (4.5%) were significant contributors to the hesitancy (*p* < 0.001 in both instances). It should be pointed out, however, that a significant proportion of the participants did not express an opinion on this matter. 

### 3.2. Factors Associated with Second-Booster Hesitancy following Multivariate Analysis

A bivariate analysis was conducted (Table 1), and factors that showed statistical significance included age, hospitalization due to COVID-19 infection, brand of the first COVID-19 booster, experience of severe/critical reactions following the first/second dose of the COVID-19 vaccine, mild reaction following the first booster, and messages heard from the government, social media/telecommunication apps, and family/close social group (*p* < 0.05). To identify predictors, factors with a *p*-value < 0.25 in the bivariate analysis were entered into the multivariate linear regression.

In the multivariate analysis (Table 2), predictors of second-booster hesitancy were identified. Age, Indian ethnicity, being a recipient of the Sinovac vaccine as the first COVID-19 booster, experiencing the death of close friends or immediate family members following COVID-19 vaccination, and receiving negative messages (indicating that taking a booster dose is harmful) from caregivers, friends, or family members were found to be associated with an increased second-booster hesitancy score. Conversely, positive messages (indicating that taking a booster is helpful) from the government and caregivers, friends, or family members were identified as predictors associated with a reduction in second booster hesitancy score.

## 4. Discussion

### 4.1. Factors Associated with Hesitancy toward a Second Booster of the COVID-19 Vaccine

This study has examined the factors that influence hesitancy toward the second booster of the COVID-19 vaccine among elderly people from residential care homes. Some recent reviews have highlighted factors frequently reported as influencing vaccine booster uptake [25,26,27]. Factors investigated include demographics of which age has been found to be more relevant in the context of vaccine booster hesitancy. However, it has been noted that the result of this association is conflicting between different studies [25]. In an extensive systematic review by Kadafar et al. [28], which looked at vaccine hesitancy in the general population, younger age was identified as one of the demographic factors positively associated with vaccine hesitancy in general. However, it does not negate our observation of the association of vaccine hesitancy with increasing age, as our study group specifically involved the elderly from residential care facilities. Hence, it is not appropriate to carry out a comparison with respect to this factor. 

Other factors often quoted across studies are adverse events following the vaccine, perceived usefulness or lack thereof of the booster, perceived level of susceptibility for COVID-19, vaccination recommendations from various sources, and the level of trust in these recommendations [25,26,27]. Of these reported factors, the only one found to be significantly associated with vaccine hesitancy in the present context, based on our multivariate analysis, was adverse events (death) among close contacts/family members post-vaccination. 

The Malaysian government mandates require the administration of both the first and second vaccine doses, the refusal of which may result in restricted mobility. The launching of the first booster, which was encouraged, resulted in fairly widespread acceptance, an observation which could be attributed to frequent news about the emergence of new variants. As the vaccination efforts progressed, many individuals, including those in social groups, reported tolerable mild reactions. However, instances of individuals succumbing to serious reactions following the COVID-19 vaccine, though rare, were also known. The awareness and fear of the potential risk of death associated with the COVID-19 vaccine could pose a strong disincentive, or more likely lead to regretting the decision to get vaccinated in the first place; this regret might influence the choice to take the optional second booster dose [29], possibly underlying our finding that there is an association between vaccine-related deaths among close contact/family members and vaccine booster hesitancy.

Secondly, negative messaging in the form of concerns about the potential harm associated with the booster shot expressed by caregivers, friends, or family members is another predictor of second-booster hesitancy among the elderly in residential care homes. This finding is particularly relevant due to the culture of collectivism which is prevalent in Malaysia, where elderly individuals residing in care homes often have limited access to information and are surrounded by individuals who often echo prevailing sentiments. Moreover, they may have a restricted understanding of the cause-and-effect relationship between the vaccine and potential health risks. In such environments, where the elderly may rely heavily on the subjective norms of those around them, the dissemination of negative information and mistrust regarding booster shots can instil fear and reluctance among listeners [30,31]. Caregivers, close friends, and family members play a crucial role in influencing the perspectives of the elderly, often holding greater sway than influential lobbies from the government and healthcare practitioners. The impact of the culture of collectivism in Malaysia accentuates the importance of these interpersonal relationships in shaping the attitudes of the elderly population in care homes towards COVID-19 booster shots.

Conversely, this study also identified that positive messages regarding the booster dose, disseminated by government officials and caregivers, friends, and family members, constitute significant factors in mitigating second booster hesitancy. In stark contrast to negative influences, the support and endorsement from these trusted sources play a crucial role in encouraging acceptance and willingness among individuals who may have initially harboured hesitancy. This is in line, with the principles of the Social Influence Theory, which posits that a person’s thoughts, feelings, and behaviours are influenced by the presence or actions of others [32]. Therefore, recognizing the impactful role of positive messaging from trusted community figures highlights the importance of targeted communication strategies to promote vaccine acceptance, particularly in the context of booster doses.

Indian ethnicity was identified as another risk factor for hesitancy, possibly due to the fact that the elderly Indian individuals in this study comprise the highest proportion (4.4%) experiencing serious adverse reactions from the first or second vaccine dose. This is coupled with the finding that this ethnic group made up a higher proportion of those who did not have chronic illness (26.7%) and were not on medication for chronic illness (31.3%) compared to the other ethnic groups [28,33,34]. In summary, the association between Indian ethnicity and vaccine hesitancy appears to be due to the combination of a higher incidence of critical reactions and possibly complacency due to the relatively low frequency of chronic illnesses, findings supported by Limbu et al. [25], in their review that highlighted adverse events, perceived susceptibility and health status as among thirteen key factors that influence booster hesitancy. 

Having Sinovac as the first booster was identified as a predictor of hesitancy towards receiving a second booster among the elderly in care homes. This could be attributed to more cases who required treatment and/or hospitalization (14.6%) and death (12.4%) among close friends and immediate family members after the first dose of Sinovac vaccine compared to the other vaccine brands. Likewise, with the second dose of Sinovac, with 15.9% needing treatment/hospitalization and 13.6% deaths. However, the first booster using Sinovac, while also associated with relatively high rates of treatment/hospitalization (14.3%) and deaths (11.9%), was second to AstraZaneca with rates of 17.4% and 21.7%, respectively. Nevertheless, mild reactions after the Sinovac booster were most common among the elderly recipients interviewed. In the present context, the common occurrence of mild reactions among Sinovac-booster recipients, coupled with reports of hospitalization and critical reactions among their close contacts (i.e., friends and immediate family members) who received Sinovac as the initial doses, could collectively contribute to the hesitancy among individuals who have previously received the Sinovac vaccine to take a second booster dose. This observation can, at least in part, be understood on the basis of social cognition constructs, highlighted by Hagger MS, et.al., 2022 [35]. The authors of this paper found that perceptions of control and risks influence vaccine acceptance, although less so compared to attitudes and subjective norms. 

### 4.2. Implications

The findings of the present study could conceivably provide information with public health significance for policymakers and healthcare practitioners. First, as increasing age among the elderly is a notable predictor of second-booster hesitancy, the development of personalized communication strategies is recommended, with reference to this high-risk group. 

Second, culturally sensitive education efforts are also relevant. Healthcare practitioners could collaborate with community leaders to enhance vaccine education and acceptance within particular ethnic groups, such as the Indian elderly community in the current context. 

Third, transparent communication from vaccine brands is important when hesitancy is tied to a particular vaccine brand. Public awareness campaigns should incorporate detailed information about various vaccine brands to foster transparency, address specific concerns, and maintain public trust. 

Fourth, empathy-infused communication and mental health support is necessary to address the emotional impact of losing loved ones and close contacts post-vaccination. Recognition of the emotional aspects of vaccine hesitancy should prompt the incorporation of mental health support services within vaccination programs. 

Fifth, given the impact of the culture of collectivism, caring for vaccine recipients with adverse outcomes should actively involve caregivers, friends, and family members. Community-focused campaigns that leverage the influence of key public figures in disseminating positive information and addressing negative sentiments could help to address and counteract factors contributing to hesitancy. 

Lastly, in the context of the elderly under residential care, collaborative efforts with elderly residential care homes represent a pivotal strategy. These initiatives could include educational campaigns, direct engagement with residents, and support from caregivers. Addressing concerns about critical reactions, potential side effects, and the emotional toll of losing close contacts post-vaccination should be central to these initiatives. 

### 4.3. Limitations and Future Directions

It is acknowledged that there are limitations within this study. First, this study is an initial examination of an evidence-based empirical study on hesitancy surrounding vaccine booster doses, an area that has received relatively little research scrutiny. The participants are elderly residents from a random sample of care homes within the Klang Valley, a geographically limited region of the country. Hence, the results are non-representative and thus do not fully capture the diversity of perspectives of the elderly from residential care homes in other regions of the country. The ethnic composition of the sample is another limitation, as the participating care homes, primarily Chinese-based, resulted in a two-third dominance of Chinese elderly participants. This imbalance may introduce selection bias and potentially affect the generalizability of the findings. Further studies, encompassing a larger and more representative sample of the elderly, would provide more comprehensive and representative data. 

Additionally, the study identifies associations between various factors and hesitancy toward the second booster. The cross-sectional nature of the research design limits the ability to infer causal relationships between identified predictors and hesitancy. Longitudinal studies would be necessary to establish a more robust understanding of causative factors. Moreover, evaluating the effectiveness of targeted interventions would provide valuable insights for public health policies and practices.

## 5. Conclusions

Amidst the global efforts against COVID-19, this study unveils a crucial aspect of vaccine acceptance within the elderly population residing in residential care homes. Despite the established efficacy of vaccines in preventing severe outcomes, the observed reluctance toward the second booster among the elderly presents a nuanced challenge. Rooted in concerns about perceived lower risk and a steadfast belief in the adequacy of the initial dose, this hesitancy highlights the need for targeted interventions. In this intricate dance with vaccine hesitancy, tailored strategies emerge as the linchpin. The implication for healthcare practitioners and the government is the necessity of context-specific approaches. While navigating the intricate landscape of vaccine hesitancy among the elderly, adopting a multifaceted approach is imperative. By comprehending and addressing the specific concerns contributing to hesitancy, healthcare providers, relevant government agencies, and residential care homes can collaboratively work to enhance vaccine acceptance. This not only aligns with the immediate goal of safeguarding the vulnerable elderly population but also contributes to broader public health endeavours in addressing the ongoing challenges posed by COVID-19.

## Figures and Tables

**Table 1:** Characteristics of participants and factors associated with second-booster hesitancy using bivariate analyses (*n* = 401).

Characteristics	Category or Details	Overall	Oxford Hesitancy Scale Mean Score (SD)	*p*-Values
Age	Mean ± SD	70.5 ± 8.1		
	Minimum–Maximum	60–97	NA	NA
	60 to 69 years	208 (51.9)	20.6 (7.2) ^a^	0.007
	70 to 79 years	123 (30.7)	22.1 (7.0) ^ab^	
	80 to 89 years	64 (16.0)	23.2 (7.0) ^ab^	
	90 and above	6 (1.5)	27.7 (4.7) ^b^	
Gender	Male	239 (59.6)	21.2 (7.2)	0.133
	Female	162 (40.4)	22.3 (7.1)	
Ethnicity	Malay	80 (20.0)	21.4 (6.1)	0.246
	Chinese	267 (66.6)	21.4 (7.5)	
	Indian	45 (11.2)	23.5 (6.9)	
	Others	9 (2.2)	19.8 (6.0)	
Education level	Primary school and below	200 (49.9)	21.9 (7.1)	>0.25
	Secondary school	167 (41.6)	21.5 (7.1)	
	University, college and above	34 (8.5)	20.5 (8.1)	
Do you have any chronic disease?	Without	79 (19.7)	21.0 (7.0)	>0.25
	With	322 (80.3)	21.8 (7.2)	
Are you on any medication for chronic disease?	Not on medication	90 (22.4)	20.9 (7.0)	>0.25
	On medication	311 (77.6)	21.8 (7.2)	
Have you been ill with COVID-19?	No	318 (79.3)	21.3 (7.1)	0.069
	Yes	83 (20.7)	22.9 (7.2)	
Have you been hospitalized due to COVID-19?	No	382 (95.3)	21.4 (7.1)	0.037
	Yes	19 (4.7)	25.0 (7.1)	
Which brand of vaccine was your first dose of COVID-19 vaccine?	Pfizer	142 (35.4)	20.8 (7.4)	0.156
	Sinovac	89 (22.2)	22.8 (6.9)	
	Astrazeneca	32 (8.0)	20.5 (9.6)	
	I don’t know	138 (34.4)	21.9 (6.3)	
Which brand of vaccine was your second dose of COVID-19 vaccine?	Pfizer	145 (36.2)	20.9 (7.5)	0.180
	Sinovac	88 (21.9)	22.8 (6.9)	
	Astrazeneca	29 (7.2)	20.3 (9.8)	
	I don’t know	139 (34.7)	21.9 (6.3)	
Which brand of vaccine was your first booster of COVID-19 vaccine?	Pfizer	186 (46.4)	20.7 (7.4)	0.029
	Sinovac	42 (10.5)	24.0 (6.8)	
	Astrazeneca	23 (5.7)	23.1 (9.5)	
	I don’t know	150 (37.4)	21.9 (6.4)	
Did you have any mild reaction to the first/second dose of COVID-19 vaccine?	No	280 (69.8)	21.9 (7.0)	0.164
	Yes	121 (30.2)	20.8 (7.5)	
Did you have any serious or critical adverse reactions from the first/second dose of COVID-19 vaccine that require treatment and/or hospitalization?	No	394 (98.3)	21.5 (7.1)	0.004
	Yes	7 (1.7)	29.3 (4.4)	
Did you have any mild reactions after the first booster of COVID-19 vaccine?	No	291 (72.6)	22.1 (7.1)	0.039
	Yes	110 (27.4)	20.4 (7.4)	
Did you have any serious or critical adverse reactions from the first booster of COVID-19 vaccine that require treatment and/or hospitalization?	No	393 (98.0)	21.6 (7.1)	>0.25
	Yes	8 (2.0)	22.8 (10.9)	
What was the top concern or worry after taking the first booster of the vaccine?	I was not concerned	329 (82.0)	21.4 (7.1)	0.119
	I was concerned that its protective effects will not last that long	11 (2.7)	18.9 (7.9)	
	I was concerned that it may cause serious long-term side effects	61 (15.2)	23.1 (7.1)	
Did any of your close friends/immediate family members need treatment/hospitalization due to the reaction following the COVID-19 vaccine?	No	285 (71.1)	21.6 (7.0)	>0.25
	Yes	33 (8.2)	23.2 (9.3)	
	No contact with friends/family	83 (20.7)	20.9 (6.8)	
Did any of your close friends/immediate family members die following the COVID-19 vaccine?	No	285 (71.1)	21.7 (6.9)	0.063
	Yes	34 (8.5)	23.6 (9.3)	
	No contact with friends/family	82 (20.4)	20.3 (6.9)	
What kind of message do you get from the information posted by the government on the COVID-19 vaccine booster?	Taking the booster dose is helpful	137 (34.2)	18.5 (7.4)	<0.001
	It is neither helpful or harmful	45 (11.2)	23.3 (6.8)	
	Taking the booster dose is harmful	2 (0.5)	28.0 (9.9)	
	I don’t know	217 (54.1)	23.1 (6.5)	
What kind of message do you get from the information posted by the social media or telecommunication apps on the COVID-19 vaccine booster?	Taking the booster dose is helpful	72 (18.0)	17.9 (8.0)	<0.001
	It is neither helpful or harmful	59 (14.7)	21.1 (6.5)	
	Taking the booster dose is harmful	18 (4.5)	22.9 (7.9)	
	I don’t know	252 (62.8)	22.7 (6.7)	
What kind of message do you get from the information posted by the caregivers, friends or family members on the COVID-19 vaccine booster?	Taking the booster dose is helpful	64 (16.0)	16.5 (7.3)	<0.001
	It is neither helpful or harmful	61 (15.2)	22.8 (6.8)	
	Taking the booster dose is harmful	45 (11.2)	25.3 (7.0)	
	I don’t know	231 (57.6)	22.0 (6.6)	
Second booster hesitancy by Oxford COVID-19 Vaccine Hesitancy Scale	Mean ± SD	21.6 ± 7.2	NA	NA
	Minimum–Maximum	7–35		

Note: A *p*-value of <0.05 was considered statistically significant for both Student *t*-test and ANOVA tests. ^a,b^ The superscript lowercase ‘a’ and ‘b’ in the table indicate subsets among various mean values. These subsets were determined using the Tukey HSD post hoc test, which was employed to identify statistically significant homogeneous groups. The same lowercase letter in the superscript denotes that the corresponding means are not significantly different from each other, while different letters indicate significant differences.

**Table 2:** Predictors of second-booster hesitancy—results of multivariate linear regression analyses (*n* = 401).

Characteristics	Indicators	B Coefficient	95% CI	*p*-Values
Constant		7.735	1.152, 14.319	0.021
Age		0.207	0.122, 0.292	<0.001
Gender (Reference: Female)	Male	−1.043	−2.397, 0.212	0.131
Ethnicity (Reference: Malay)	Chinese	−1.193	−2.892, 0.506	0.168
	Indian	2.554	0.219, 4.889	0.032
	Others	−1.400	−5.829, 3.029	>0.25
Have you been ill with COVID-19? (Reference: No)	Yes	0.511	−1.276, 2.299	>0.25
Have you been hospitalized due to COVID-19? (Reference: No)	Yes	1.458	−1.844, 4.760	>0.25
Which brand of vaccine was your first booster of COVID-19 vaccine? (Reference: I don’t know/Others)	Pfizer	−0.446	−1.878, 0.986	>0.25
	Sinovac	3.440	1.110, 5.770	0.004
	Astrazeneca	1.584	−1.279, 4.447	>0.25
Did you have any mild reaction to first/second dose of COVID-19 vaccine? (Reference: No)	Yes	0.108	−1.495, 1.710	>0.25
Did you have any serious or critical adverse reactions from the first/second dose of COVID-19 vaccine that require treatment and/or hospitalization? (Reference: No)	Yes	4.206	−0.818, 9.230	0.101
Did you have any mild reactions after the first booster of COVID-19 vaccine? (Reference: No)	Yes	−1.076	−2.729, 0.576	0.201
What was the top concern or worry after taking the first booster of the vaccine? (Reference: I was not concerned)	I was concerned that its protective effects will not last that long	−2.619	−6.568, 1.329	0.193
	I was concerned that they may cause serious long-term side effects	0.981	−0.922, 2.883	>0.25
Did any of your close friends/immediate family members die following the COVID-19 vaccine? (Reference: No contact with friends/family)	No	1.636	−0.067, 3.338	0.060
	Yes	3.413	0.748, 6.079	0.012
What kind of message do you get from the information posted by the government on the COVID-19 vaccine booster? (Reference: I don’t know)	Taking the booster dose is helpful	−3.232	−5.016, −1.448	<0.001
	It is neither helpful or harmful	1.095	−1.277, 3.467	>0.25
	Taking the booster dose is harmful	0.997	−8.775, 10.769	>0.25
What kind of message do you get from the information posted by the social media or telecommunication apps on the COVID-19 vaccine booster? (Reference: I don’t know)	Taking the booster dose is helpful	−0.917	−3.143, 1.309	>0.25
	It is neither helpful or harmful	−1.919	−4.100, 0.262	0.085
	Taking the booster dose is harmful	−0.984	−4.298, 2.330	>0.25
What kind of message do you get from the information posted by the caregivers, friends or family members on the COVID-19 vaccine booster? (Reference: I don’t know)	Taking the booster dose is helpful	−3.191	−5.310, −1.072	0.003
	It is neither helpful or harmful	1.834	−0.201, 3.869	0.077
	Taking the booster dose is harmful	3.328	1.128, 5.528	0.003

Notes: 1. The questions, “Which brand of vaccine was your first dose of COVID-19 vaccine?” and “Which brand of vaccine was your second dose of COVID-19 vaccine?” were initially included in the multivariate linear regression analysis as they met the selection criteria (*p*-value < 0.25). However, they were subsequently excluded from the analysis due both to violation of the assumption of multicollinearity and that both variables exhibited a Variance Inflation Factor (VIF) exceeding 10 and a Tolerance below 0.1. Therefore, in adherence to multicollinearity considerations, these variables were removed from the regression model. 2. The *p*-value in the ANOVA output is < 0.001 (significance cut off point is *p* < 0.05), indicating that the multivariate model is well-suited for assessing second-booster hesitancy. The R-squared value of 0.304 signifies that 30.4% of the variation in the second booster hesitance score can be explained by the factors included in the model. Autocorrelation is not present in the model, as evidenced by a Durbin–Watson value of 1.795 (preferred range: 1.0–3.0). Furthermore, there is no issue of multicollinearity, as all variables fall within the desirable thresholds; the Variance Inflation Factor (VIF) is <10, and Tolerance is >0.1. No outliers are identified in the current dataset based on the definition of outliers as any number with a standard deviation of 3 units. Regarding homoscedasticity, the data exhibits a consistent scatter without any discernible pattern, and the residuals are normally distributed. 3. A *p*-value of <0.05 was considered statistically significant in multivariate linear regression analyses.

## Data Availability

The data underlying this article are available in the article.
